# Supercritical Fluid Extraction of Pyrrolidine Alkaloid from Leaves of* Piper amalago* L.

**DOI:** 10.1155/2017/7401748

**Published:** 2017-05-02

**Authors:** V. S. Carrara, L. C. Filho, V. A. S. Garcia, V. S. Faiões, E. F. Cunha-Júnior, E. C. Torres-Santos, D. A. G. Cortez

**Affiliations:** ^1^Department of Chemical Engineering, State University of Maringá, 87020-900 Maringá, PR, Brazil; ^2^Laboratory of Biochemistry of Trypanosomatids, Oswaldo Cruz Institute, FIOCRUZ, 21040-360 Rio de Janeiro, RJ, Brazil; ^3^Department of Pharmacy, State University of Maringá, 87020-900 Maringá, PR, Brazil

## Abstract

Supercritical fluid extraction was used to extract the alkaloid* N*-[7-(3′,4′-methylenedioxyphenyl)-2(*Z*),4(*Z*)-heptadienoyl]pyrrolidine from leaves of* Piper amalago* L. A three-level orthogonal array design matrix, OAD OA_9_(3^4^), was used for optimization of the parameters of supercritical extraction of the alkaloid, employing supercritical carbon dioxide: extraction time (20, 40, and 60 min), temperature (40, 50, and 60°C), pressure (150, 200, and 250 bar), and the use of cosolvents (ethanol, methanol, and propyleneglycol). All parameters had significant effect on the alkaloid yield. The alkaloid yield after 60 min of extraction without cosolvents at 9 different conditions (3^2^) in terms of temperature (40, 50, and 60°C) and pressure (150, 200, and 250 bar) was also evaluated. The optimal yield (≈3.8 mg g^−1^) was obtained with supercritical CO_2_ + methanol (5% v : v) at 40°C and 200 bar for 60 min of extraction.

## 1. Introduction

*Piper amalago* L. (Piperaceae) is a shrub that spans a height of 2–7 m, distributed from Mexico to the south of Brazil. Popularly known as jaborandi-manso, it is traditionally used to treat heart problems, like hypertension, burns, inflammation [1, 2], and infections [3, 4]. The infusion of leaves is typically used to relieve intestinal colic [5], stomachaches [3], and muscle aches [6]. The alcoholature of the leaves is used during the bath to hydrate and treat the loss of hair [6]. The infusions of the roots are used as diuretic and against renal stones [5]. Pharmacological investigations have also shown that the extracts from* P. amalago* leaves present anti-inflammatory [7], anxiogenic [8], diuretic, natriuretic, antilithiatic [9], healing [10], schistosomicidal [11], and antileishmanial activities [12].

The chemical components recently found in leaves of this plant are (**1**)* N*-[7-(3′,4′-methylenedioxyphenyl)-2(*Z*),4(*Z*)-heptadienoyl]pyrrolidine, (**2**)* N*-[7-(3′,4′-methylenedioxyphenyl)-2(*E*),4(*E*)-heptadienoyl]pyrrolidine [12], (**3**) N-[3-(6′-methoxy-3′,4′-(methylenedioxyphenyl)-2(Z)-propenoyl]pyrrolidine, (**4**) N-[3-(6′-methoxy-3′,4′-methylenedioxyphenyl)-2(*E*)-propenoyl]pyrrolidine [10], (**5**) lupeol, and (**6**) vitexin [13].

The compound** 1*** N*-[7-(3′,4′-methylenedioxyphenyl)-2(*Z*),4(*Z*)-heptadienoyl]pyrrolidine has showed important antifungal [14], antileishmanial [15], and schistosomicidal activities [11] and has been found in considerable quantity in leaves of* P. amalago *L. The alkaloid 2 showed antileishmanial activity. Pyrrolidine alkaloids** 1** and** 2**, which have been related to benefic effects of* P. amalago* L., are soluble in chloroform and dichloromethane, which are toxic to the health human and environment. Therefore, the extraction using clean technology is indispensable. In this sense, supercritical fluid extraction is safer for the human being, due to the reduction of the volume of organic solvent and extraction time [16]. Authors have been extracting alkaloids semipurified by supercritical fluid extraction [17–19], and we have extracted the pyrrolidine alkaloids with better yield than chloroform [12]. Considering the important biological activities of compound** 1**, it is indispensable to evaluate the factors which influence its extraction using supercritical carbon dioxide.

Therefore, the objective of the present work was to identify and evaluate the previous operational parameters involved in the process of the extraction of alkaloid** 1** from leaves of* P. amalago* L. using supercritical carbon dioxide. The methodology used was a four-level three factor orthogonal array design (OAD) with an OA_9_(3)^4^ matrix, considering the following parameters: cosolvents, time of extraction, temperature, and pressure [20, 21]. Moreover, extractions employing only carbon dioxide CO_2_ using the planning by blocking were also studied.

## 2. Materials and Methods

### 2.1. Plant Material

*Piper amalago* L. leaves (2 kg) were collected from the Bosque II, Maringá, Paraná, Brazil, whose voucher specimen (number HUEM 9885) was deposited in the herbarium of the Department of Botany, State University of Maringá. Leaves were dried in an air circulating oven (Quimis®, model Q-31) for three days and crushed in a knife grinder (Tecnal Marconi®, model TE 048, Piracicaba, Brazil). Particles with medium diameter of 0.757 mm were used for preparation of the extracts using supercritical carbon dioxide with or without cosolvents.

### 2.2. Purification of the Alkaloid

The isolation of the alkaloid N-[7-(3′,4′-methylenedioxyphenyl)-2(Z),4(Z)-heptadienoyl]pyrrolidine (compound** 1**) from* P. amalago* L. leaves and its spectral data were described in a previous study [12].

### 2.3. Extraction Using Supercritical Carbon Dioxide

The experiments were performed with the following equipment: CO_2_ reservoirs (both technical grade obtained from White Martins®, Rio de Janeiro, Brazil), two thermostatic baths, a syringe pump (Teledyne Isco®, model 500D, Lincoln, USA) and an extractor with dimensions of 17 × 2 cm, an absolute pressure transducer (Smar®, model LD 301, São Paulo, Brazil) equipped with a portable program (Smar, model HT 201, São Paulo, Brazil) with an accuracy of ±0.031 MPa, a micrometric valve, and amber glass bottles as collectors. The extractor was loaded in a random way with approximately 12 g of noncompact powdered sample. The extraction parameters of compound were performed by orthogonal array design (OAD) OA_9_(3^4^) (Table 1) [16]. In the present work, four factors were studied by a three-level OAD: modifiers, dynamics extraction time, temperature, and pressure. The effects of the parameters on the yield of compound** 1** were studied by analysis of variance (ANOVA). The flow rate used for the extractions was 3 mL/min with 5% of cosolvent (v/v). The powder particle diameter was 0.757 mm. All the conditions were realized in triplicate. Extractions employing only CO_2_ were also studied using the planning by blocking, according to Table 2, with the central point 50°C, 200 bar. The flow rate of CO_2_ was 3 mL/min and the time of dynamic extraction was 60 min. All the experiments were done in triplicate.

### 2.4. HPLC Analysis

Extracts were analyzed by High Performance Liquid Chromatography, according to a validated method. The equipment used was as follows: using a Gilson 321 Binary HPLC Pump (Middleton, WI, USA) equipped with a manual injection valve with a loop of 20 *μ*l, Gilson 864 degasser, and a Gilson 152 UV/visible detector (Middleton, WI, USA), controlled by Borwin version 1.5 Software (Easton, MD, USA). The chromatographic analysis was carried out in a Kinetex C18 column (150 × 4,6 mm, d.i.) Phenomenex®, packed with 5 *μ*m particles at 25°C. The mobile phase used was 58% of acetonitrile and 42% of water containing 1% of acetic acid for 6 min, changing to 100% of acetonitrile at 7 min to 11 min, returning to 58% of acetonitrile and 42% of water at 11 min to 12 min, at flow rate of 1 ml/min. The alkaloid was detected at 260 nm. The solutions of the extracts were prepared in acetonitrile at 300 *μ*g/ml and filtered through a nonsterile 0.45 *μ*m membrane filter (Millipore®, São Paulo, Brazil). A volume of 20 *μ*l of each sample was manually injected into the HPLC, and the analysis was carried out in triplicate. The data were evaluated by the software Statistica® 6.0. In order to evaluate the alkaloid content in the extracts, a curve of calibration was carried out by the external standard method, using alkaloid** 1** isolated previously as standard. A stock standard solution of 1000 *μ*g/ml in acetonitrile was prepared and diluted to the following concentrations: 15, 45, 100, 150, and 200 *μ*g/ml. The analyses were carried out in triplicate. The calibration function was *y* = 30344 + 63869. The coefficient of determination for the linear regression was calculated as *r*^2^ = 0.999. The relative standard deviation was lesser than 5% and the accuracy was above 98% for all concentrations.

## 3. Results and Discussion

### 3.1. Chromatograms Obtained

**Figure 1:**
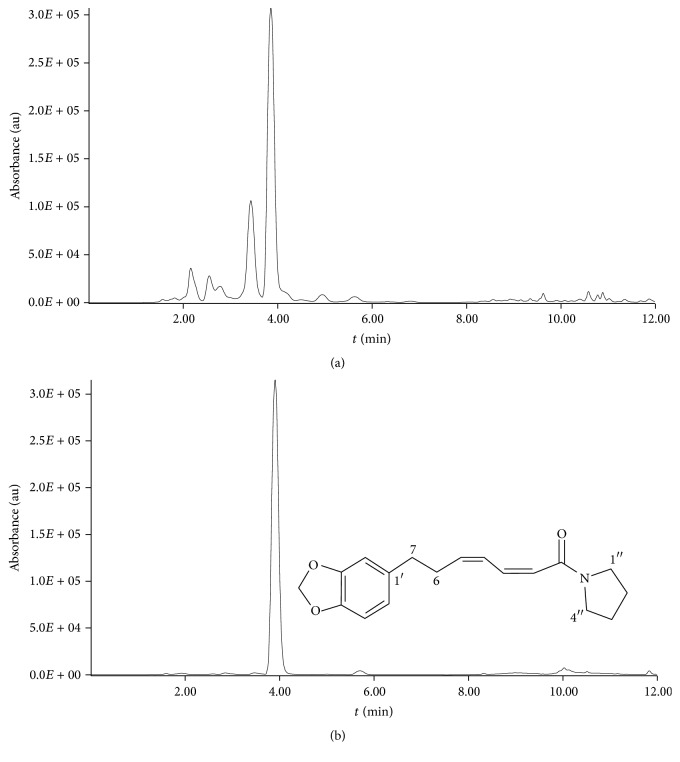
(a) Chromatogram of an extract obtained by supercritical CO_2_ + 5% methanol (v : v) at 40°C, 200 bar for 60 min. (b) Chromatogram of the examined alkaloid whose retention time was 4 min. Chromatograph conditions: column Kinetex C18 column Phenomenex (150 × 4.6 mm, d.i.), at 25°C; mobile phase used was 58% of acetonitrile and 42% of water containing 1% of acetic acid, changing to 100% of acetonitrile at 7 min to 11 min, returning to 58% of acetonitrile and 42% of water at 11 min to 12 min, flow rate of 1 ml/min; temperature at 25°C; detection at 260 nm.

Carbon dioxide and cosolvents at supercritical conditions were used to have extracts from* P. amalago* L. leaves containing the pyrrolidine alkaloid. As already explained, the purpose was to evaluate the effect of factors, which are usually important in an operation of extraction, on alkaloid yield. An example of chromatogram that reveals the presence of the pyrrolidine alkaloid in the examined extracts and the chromatogram of a standard solution with pure pyrrolidine are presented in Figure 1.

### 3.2. Study of the Operational Parameters Involved in the Extraction of Alkaloid** 1** by OAD OA_9_(3)^4^

**Figure 2:**
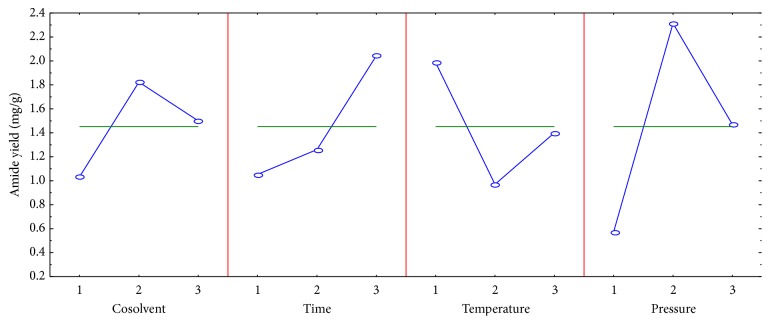
The effect of the parameters on the alkaloid yield. Cosolvent (1 = ethanol, 2 = methanol, and 3 = propyleneglycol); time (1 = 20 min, 2 = 40 min, and 3 = 60 min); temperature (1 = 40°C, 2 = 50°C, and 3 = 60°C); pressure (1 = 150 bar, 2 = 200 bar, and 3 = 250 bar). The error bar shows standard deviations for *n* = 3.

The experimentation planning and the data obtained for the alkaloid extraction are shown in Table 1. The data were analyzed using Statistica 6.0 program for evaluating the effect of each parameter on the extraction of the compound** 1**. The data of the analysis of variance (ANOVA) of this study are shown in Table 2. The relation between the yield of the alkaloid and the different factors is shown in Figure 2.

The amount of the compound** 1** (2.03 mg·g^−1^ of the plant) was also evaluated in the chloroform extract, which was obtained by the remaceration method.

The effect of cosolvents was evaluated. Considering the alkaloid an intermediate polarity compound, ethanol, methanol, and propyleneglycol were used as polar modifiers in the extraction by supercritical CO_2_, with the view to verify the effect of each one on the alkaloid yield. The cosolvents related have been shown to improve the efficiency of the alkaloids extraction and reduce the extraction time. According to the results shown in Figure 2 and Table 1, the yield of the alkaloid was significantly affected by the change of the modifiers. The mean yield varied from 1 to 2 mg·g^−1^, with the minimum value obtained with ethanol and the maximum when using methanol as cosolvent. Other authors also concluded that the methanol was the best cosolvent used to improve the yield of the extraction of the alkaloids and amides [22–24]. The yield of the extraction of the compound** 1** (2 mg·g^−1^) was similar to that obtained by chloroform extraction. However, the supercritical fluid extraction employing methanol as cosolvent used less solvent.

The time of extraction (Figure 2 and Table 2) influenced significantly the yield of the alkaloid, which varied around from 1 to 2.05 mg·g^−1^, obtaining the highest yield in 60 min. Therefore, it takes about an hour to obtain the analyte of the interest with efficiency, without overspending wasting. Studies about supercritical fluid extraction of the alkaloids and amides using carbon dioxide have shown a time of 60 min to obtain an optimal yield of the analytes [19, 23, 25]. The quantity of alkaloid** 1** extracted for 60 min was similar to the yield obtained by the remaceration method for three days. Considering that the efficiency of the extraction is related to quantity of the solvent and time employed, compound** 1** was extracted with more effectiveness in less time.

Figure 2 and Table 2 showed the effect of the temperature on the yield of the alkaloid. The yield varied at range from 0.96 to 2.00 mg·g^−1^, with the temperature of 40°C being the most appropriate to extract the analyte. The increasing of the temperature to 50°C led to the lowest yield. Compound** 1** was extracted in medium conditions compared to the other alkaloids extracted at higher temperatures [19, 26–29]. The study showed that temperature higher than 25°C is needed to extract alkaloid** 1** with the similar yield of the compound obtained by the chloroform extraction.

Different values of pressure, 150, 200, and 250 bar, were employed to analyze the effect of the pressure on the yield to the alkaloid. Figure 2 and Table 2 showed that the pressure influenced the alkaloid yield significantly, with the mean ranging between 0.70 and 2.30 mg·g^−1^. The analyte was extracted with the increasing of the pressure, obtaining the highest yield with the pressure of 200 bar. The yield of the extraction of compound** 1** (2.30 mg·g^−1^) was better than yield of the compound obtained by the chloroform extraction. Alkaloids have been extracted with temperature and pressure above 50°C and 200 bar, respectively [19, 26–29]. In the present work, the pyrrolidine alkaloids were extracted with mild conditions of temperature and pressure (40°C, 200 bar). Therefore, the densities of the carbon dioxide were according to the literature.

### 3.3. Study of the Operational Parameters Involved in the Extraction of Alkaloid** 1** by Planning for Blocking

In order to certify if the modifier contributed to the efficiency of the extraction, nine conditions of extractions were studied, using supercritical CO_2_ in the absence of cosolvents under different temperatures and pressures. The planning by blocking was used for the experiments, with the central point (50°C, 200 bar) carried out in triplicate. The flow rate of supercritical CO_2_ was about 3 mL/min, and the time of extraction was 60 min in the conditions.

The yield of alkaloid 1 at such conditions is presented in Table 3. The data showed that the yield of the alkaloid was higher (1.11 ± 1.60 mg/g) at 60°C, 250 bar, and 60 min of extraction. All extractions using only supercritical CO_2_ led to less yields of the alkaloid than the extractions employing supercritical CO_2_ more cosolvents. Alkaloids are compounds with intermediate polarity and have little solubility in carbon dioxide. Modifiers must be added to improve the extraction, and methanol was the better cosolvent in this study.

Therefore, the condition of extraction that resulted in the highest alkaloid content (*Y*_Ap_ = 3.8 ± 0.8 mg g^−1^) was supercritical CO_2_ at 40°C, 200 bar, cosolvent methanol 5%, 60 min of extraction, and flow rate of 3 mL/min. Additionally, the extraction efficiency of compound** 1** obtained was higher than that obtained by chloroform extraction.

## 4. Conclusions

Different conditions of extraction were employed with the objective of studying the effect of them on the alkaloid yield of* P. amalago* L. All parameters affect the alkaloid yield. Cosolvents contributed significantly with the efficiency of the extraction of the alkaloid. Results indicated that the highest alkaloid content (run 6 in Table 1, *Y*_Ap_ = 3.8 ± 0.8 mg g^−1^) was obtained employing the following extract conditions: the higher time (60 min) of extraction and mild conditions of temperature and pressure (40°C, 200 bar) and methanol as cosolvent. To the best of our knowledge, this is the first study about the extraction parameters of the pyrrolidine alkaloid from this plant. The present work may be used with reference to the additional studies to discover the optimal extraction conditions of all alkaloids in the plant.

## Figures and Tables

**Table 1:** Orthogonal array design matrix OAD OA_9_(3)^4^ and experimental results of alkaloid **1** yield.

Run	Factors				Responses
	Cosolvent	*t* (min)	*T* (°C)	*P* (bar)	*Y*_Ap_ ± *σ*_p_ (mg g^−1^) (*n* = 3)
1	Ethanol	20	40	150	0.29 ± 0.02
2	Ethanol	40	50	200	1.2 ± 0.3
3	Ethanol	60	60	250	1.59 ± 0.07
4	Methanol	20	50	250	1.0 ± 0.3
5	Methanol	40	60	150	0.7 ± 0.9
6	Methanol	60	40	200	3.8 ± 0.8
7	Propyleneglycol	20	60	200	1.9 ± 0.3
8	Propyleneglycol	40	40	250	1.86 ± 0.08
9	Propyleneglycol	60	50	150	0.73 ± 0.01

*Y*_Ap_ = yield of the alkaloid in the plant.

Results are expressed as mean ± standard deviation (*σ*).

**Table 2:** Analysis of variance (ANOVA) of four parameters for the extraction using supercritical CO_2_ and cosolvents.

	Sum of squares	Degrees of freedom	*F*-ratio	*F*_0.05_	*p* value	Effect
Cosolvent	2.3	2	1.1	7.4	0.01	Significant
Time	3.1	2	1.5	10.0	0.005	Significant
Temperature	3.1	2	1.6	10.0	0.005	Significant
Pressure	8.3	2	4.1	26.6	0.0002	Significant
Residual	1.4	9	0.15			

**Table 3:** Alkaloid yield for extraction without cosolvents (only CO_2_) at different pressures and temperatures (*t* = 60 min).

Run	Factors		Responses
	*T* (°C)	*P* (bar)	*Y*_Ap_ (mg g^−1^) ± *σ*_Rp_ (%)
10	40	150	0.42 ± 1
11	50	150	0.54 ± 2
12	60	150	0.48 ± 2
13	40	200	0.97 ± 3
14	50	200	0.90 ± 12
15	60	200	1.04 ± 2
16	40	250	0.75 ± 2
17	50	250	0.71 ± 1
18	60	250	1.11 ± 2

*Y*_Ae_ = yield of the alkaloid in the extract. *Y*_Ap_ = yield of the alkaloid in the plant.

Results are expressed as mean ± relative standard deviation (*σ*_R_).
